# Supplementary material for: Whole genome sequence and comparative analysis of Borrelia burgdorferi MM1
Source: PLoS One. 2018 Jun 11;13(6):e0198135. doi: 10.1371/journal.pone.0198135 (PMC5995427; doi:10.1371/journal.pone.0198135)
Supplement: S1 Fig — An initial mapping of MM1 reads to the type strain B31 suggested lack of plasmids lp21, lp28-1, lp28-2, lp38, lp56 and lp5 in MM1 genome, while the presence/absence status of other plasmids in MM1 remained ambiguous. B31 plasmids are shown in each plot with X-axis and Y-axis representing Reference Start Position and Coverage respectively. (PDF) [file pone.0198135.s001.pdf]

Supplemental Figure 1

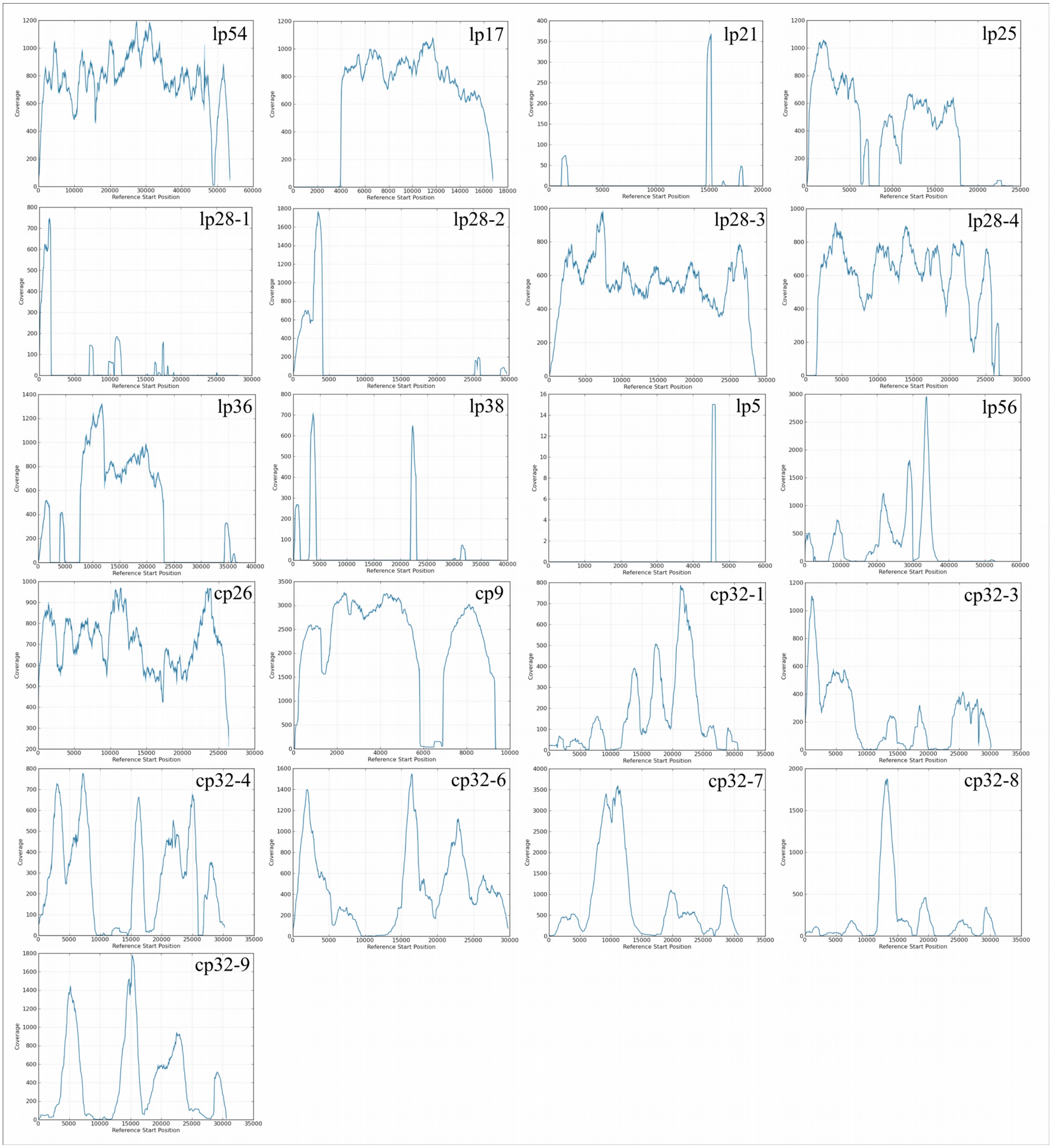

Coverage of MM1 Sequence Reads Across *B. burgdorferi* B31 Plasmids. An initial mapping of MM1 reads to the type strain B31 suggested lack of plasmids lp21, lp28-1, lp28-2, lp38, lp56 and lp5 in MM1 genome, while the presence/absence status of other plasmids in MM1 remained ambiguous. B31 plasmids are shown in each plot with X-axis and Y-axis representing Reference Start Position and Coverage respectively.
