# Supplementary figures and images for: Whole genome sequence and comparative analysis of Borrelia burgdorferi MM1
Source: PLoS One. 2018 Jun 11;13(6):e0198135. doi: 10.1371/journal.pone.0198135 (PMC5995427; doi:10.1371/journal.pone.0198135)

**A)**

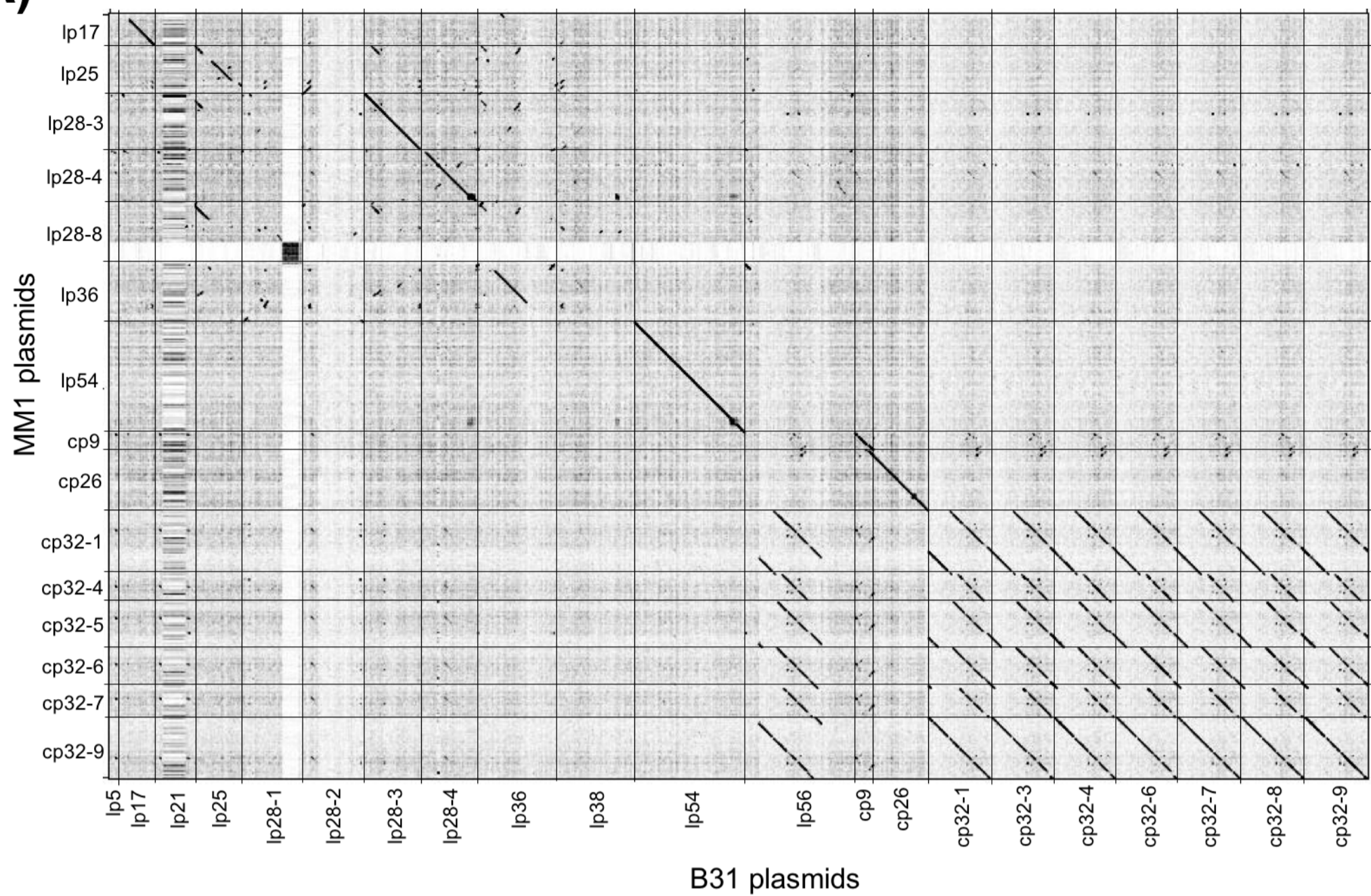

**B)**

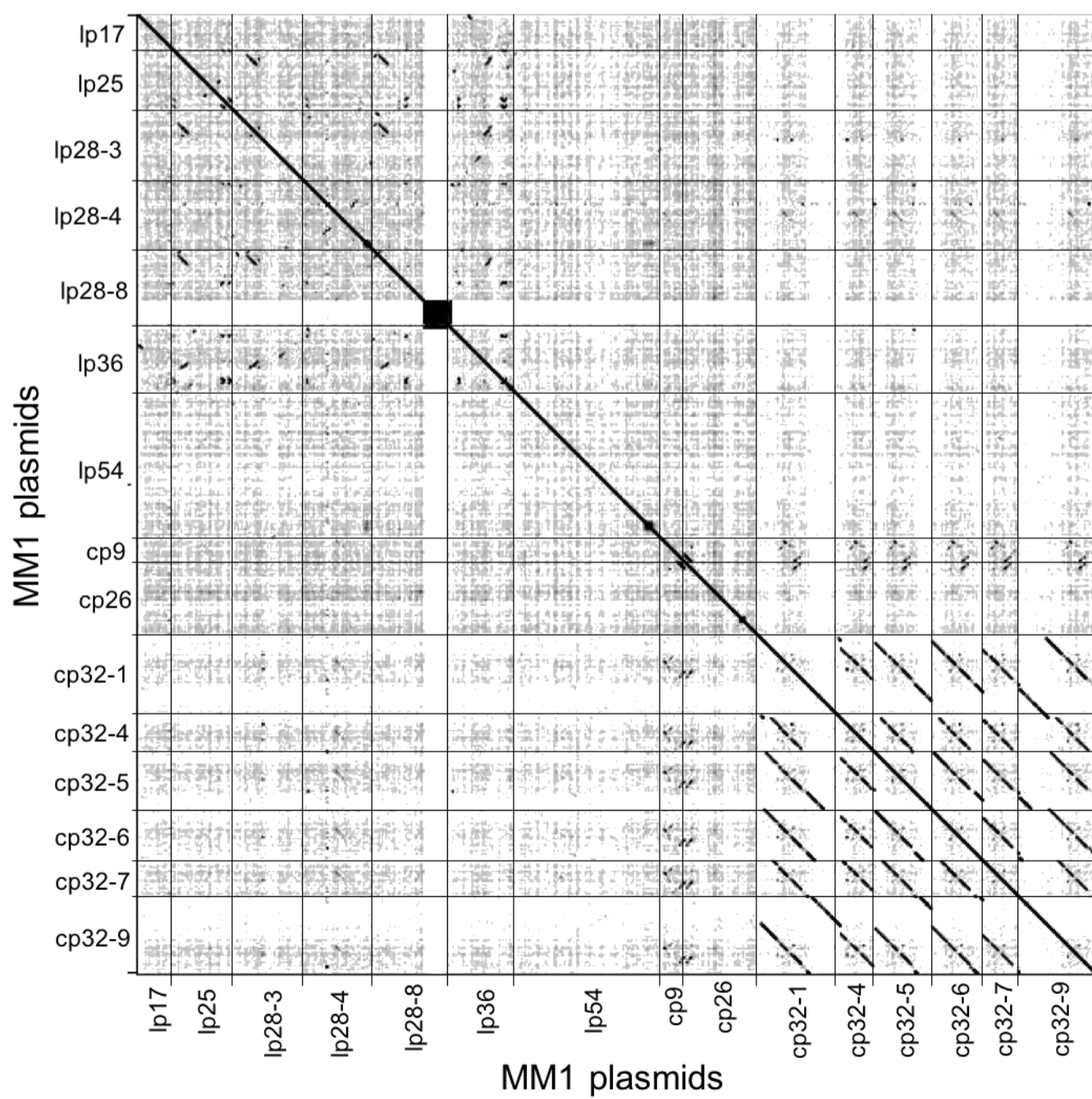

Supplement: S2 Fig — A) Dotplot of MM1 versus B31 shows a very high degree of similarity. MM1 contains a subset of the plasmids of B31. B) Dotplot of MM1 versus itself illustrates two characteristics of Borrelia genomes. First, the cp32 family of plasmids show great similarity. Second, a large repetitive region, the vls locus, appears as a prominent dark square on plasmid lp28-8. (PDF) [file pone.0198135.s002.pdf]
