# Supplementary material for: Whole genome sequence and comparative analysis of Borrelia burgdorferi MM1
Source: PLoS One. 2018 Jun 11;13(6):e0198135. doi: 10.1371/journal.pone.0198135 (PMC5995427; doi:10.1371/journal.pone.0198135)
Supplement: S3 Fig — Top: The de novo assembly of the nifS gene matches the MLST nifS type 12 except for a C->T polymorphism. Eleven nanopore reads align to this region and all contain the T variant. Bottom: The de novo assembly of the pepX gene matches the MLST pepX type 1 except for a T->C polymorphism. Nine nanopore reads align to this region and eight contain the C variant. The two SNPs of interest are noted with a red asterisk (*). (PDF) [file pone.0198135.s003.pdf]

Supplemental Figure 3

```
Name                 Sequence
MLST_nifS-12         AAAATAGAAAAAACAAGAGAACTTGTAAAAAATTTCATTAATGCAGAATCTGCAAAAAAT
Assembled_MM1_nifS   AAAATAGAAAAAACAAGAGAACTTGTAAAAAATTTTATTAATGCAGAATCTGCAAAAAAT
05e484a1-7b54-48fb   AAATAGAAAAACAAGAGAAC-----TTGTAAAATTTTTATTAAT-GAATCTGCAAA--AT
2e08a83a-bc8f-4976   AACCCGAAAAACA-----AGAGAACTTATAAAATTTATTAATGCAGAATCTGCAAAATAT
88624a5a-f257-4af9   AAAGCCGAAAACAAGA--GAAC--TTGTAAAATTTTATTAATGCAGAATCTGCA-AATAT
ebeaba4b-a7f2-4c91   AAATAGAAAAACAAGAGAAC-----TTGTAAATTTTATTAATGCAGAACTCTGCAAAATA
a753454d-707d-4b83   AAATAGAAAACAAGGGGACT-----GTAAATT--TTATTAATGCAGAAT-----------
2cdd8d93-15cd-449d   CAAAATAGAGCTAAGAA--------TTGACAATTTTGACTGCTTGGAGTCTGCAAAATAA
5a187c5f-d9c6-40d1   AAATAGAAAAACAAGAGAAC-----TTAAAAATTTTATTAATGTGCAGAATCTGCAAAA-
c5dc593f-c1fa-40f8   AAAGGCGAAAACAAGAGAAC-----TTGAAAATTTTATTAATGCAGAATCTGCAAAATAT
c3fed319-9ceb-4396   AAATAGTAGAAAAACAGGGGACTTGTAAAATTTTCTCTTAATGCAGAATCTGCAAAATAT
e7fb0bcb-85cb-4a2a   AAAATAGAAAACAAGAGAAC-----TGCTAAATTTTATTAATTGCAGAATCTG-------
0177d47f-9cdd-4c4e   AAATAGAAAAACAAGAGAAC-----TTGTAAAGTTTATTAATGCAGAATCTGCAAAA---
                                                        *
```

```
Name                 Sequence
MLST_pepX-1          TACAATGCTCATGACAATTTAACA----GTAATCAGTAGCACTAAAAAAGCAATAAAAGA
Assembled_MM1_pepX   TACAATGCTCACGACAATTTAACA----GTAATCAGTAGCACTAAAAAAGCAATAAAAGA
817a604f-2391-44b1   ACAATGCGGCACGACAATTTAACA----GTAATCAGTAGCACTAAAAGCAATAAAGATAA
a216f821-fd7a-4ebb   ATACATGCTCACGACAATTTAACG----ATGAAGATCAGCACTAAAAAGCAATAAAAGAT
aa375ec3-643a-4f7e   -TACAATTCAAGGACAATTTAACA----GTAATGAGTAGCACTAAAGCAATAAAATAATA
26deeb2d-2462-48ce   TACAATGCTCACG-ACAATTAACA----GCAATCAGTAGCACTAAAAGCAATAAAAGATA
D6a5329d-8245-4941   -ACCAATTCCACGACATTTGAATAGGTACGTATTAGTAGCACTAAAGCAATAGACAGATA
6e1fdff6-2933-419d   TACAATGCTCACGACAATTTAAA------TGATCAGTAGCACTAAAAGGGCAATAAAGAT
cfa9c429-2b73-4daf   TACAATGCTCACGACAATTTAACA----GTAATGAGTAGCACTAAAAGCAATAAAAGATA
b4a50839-4edc-485b   ACATCCTCTCACGACAATTAACAG----TAAACCAGTAGCACTCAAAAAGCAATAGAATA
64f1cd9f-45c5-47a7   TACAATGCTCACG-ACAATTAACA----GTAATCAGTAGCACTAAAACTAATAAAAGATA
                                *
```

Nanopore sequencing reads confirm the novel SNPs in *nifS* and *pepX*. Top: The de novo assembly of the *nifS* gene matches the MLST *nifS* type 12 except for a C->T polymorphism. Eleven nanopore reads align to this region and all contain the T variant. Bottom: The de novo assembly of the *pepX* gene matches the MLST *pepX* type 1 except for a T->C polymorphism. Nine nanopore reads align to this region and eight contain the C variant. The two SNPs of interest are noted with a red asterisk (*).
